# Temporal dynamics of viral load and false negative rate influence the levels of testing necessary to combat COVID-19 spread

**DOI:** 10.1038/s41598-021-88498-9

**Published:** 2021-04-28

**Authors:** Katherine F. Jarvis, Joshua B. Kelley

**Affiliations:** Department of Molecular and Biomedical Sciences, University of Maine, 5735 Hitchner Hall, Orono, ME 04469 USA

**Keywords:** Computational models, Viral infection, Public health, Epidemiology, Population screening

## Abstract

Colleges and other organizations are considering testing plans to return to operation as the COVID-19 pandemic continues. Pre-symptomatic spread and high false negative rates for testing may make it difficult to stop viral spread. Here, we develop a stochastic agent-based model of COVID-19 in a university sized population, considering the dynamics of both viral load and false negative rate of tests on the ability of testing to combat viral spread. Reported dynamics of SARS-CoV-2 can lead to an apparent false negative rate from ~ 17 to ~ 48%. Nonuniform distributions of viral load and false negative rate lead to higher requirements for frequency and fraction of population tested in order to bring the apparent Reproduction number (Rt) below 1. Thus, it is important to consider non-uniform dynamics of viral spread and false negative rate in order to model effective testing plans.

## Introduction

As schools consider their return to normal classes, they are relying on the use of tests to combat COVID-19 transmission^1^. With little information about how COVID-19 will spread through schools, decision-makers are turning to models of viral spread to estimate the amount of testing and the testing frequency required to allow a normal return to schools, as well as other interventions^2–4^.

**Figure 1:**
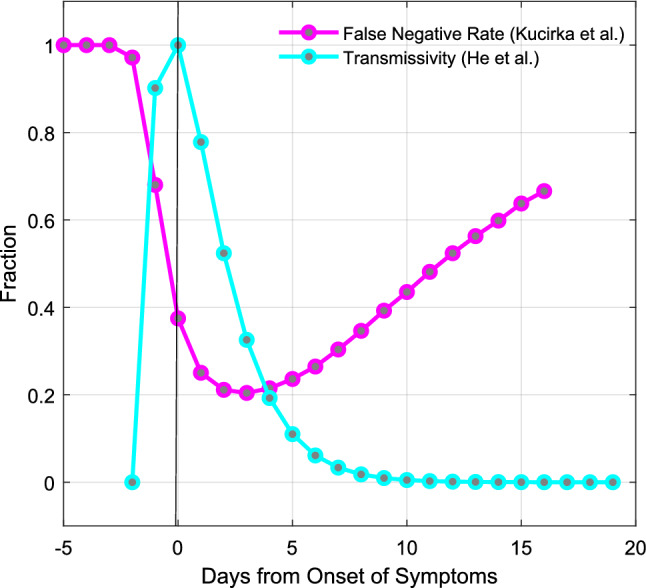
Viral transmission data and test false negative rate data both suggest that SARS-CoV-2 is undetectable until ~ 2 days prior to symptom onset. Shown in cyan is the viral load data by day from onset of symptoms from He et al.^6^. Shown in magenta is the false negative rate of tests by day from Kucirka et al.^7^. Transmission probability begins increasing ~ 2 days before symptom onset, at the same time that the false negative rate of tests begins dropping.

Central to the efficacy of mathematical models is the choice of the parameters in those models that describe the spread of the disease. In order to model testing, the model must make assumptions about how long after infection a virus is present at a level that can be detected as well as frequency of the false negative rate. Considerations about the rate of transmission of disease are also important because high levels of transmission prior to symptom onset make it harder to control the outbreak^5^. Both detection of virus by a PCR based test and transmission of disease to another person are processes that should be proportional to viral load in the patient because the presence of virus in the patient serves as the infectious agent and as the template for the test. Transmission probability relative to the date of symptom onset has been estimated by He et al. based on the serial interval of multiple transmission pairs^6^. They found that transmission probability likely starts rising just over two days before symptom onset, and that ~ 44% of transmission may occur prior to symptom onset^6^ (Fig. 1).

Assessing the efficacy of tests relies upon understanding the false negative rate of testing. The false negative rate of testing can be broken down into two basic types of false negative, one is a technical failure where the test fails on a sample with detectable levels of virus. Another type is a false negative due to the latent period of the virus, where there is not yet sufficient viral titer in the sample for it to be detected by the test. The transmission and viral load data from He et al. would suggest that prior to 2.4 days before symptom onset, infected people may not have sufficient virus to be detectable by a test. In a study by Kucirka et al., the dynamics of false negative rate over time was determined by examining data on false negative test in patients who were eventually found to be positive^7^. False negative rates were found to be 100% until two days prior to symptom onset and they reached a minimum of approximately 25% two days after symptom onset (Fig. 1).

These two studies represent two different data sets that can inform assumptions about viral load, as the ability to transmit disease and detect infection are both likely to be proportional to viral load. While He et al. estimated transmission rates from transmission pairs and compared that to measured viral loads, Kucirka et al. measured the likelihood of a positive test relative to symptom onset and collected data points from presymptomatic patients. The data from both studies predict that detection and viral spread are likely to begin approximately 2 days before symptom onset.

The ability of testing to slow the spread of disease is related to the accuracy and function of the test but also to how fast the disease spreads. In order to stop disease spread, each infected person must, on average, infect less than one other person (an effective Reproduction number (Rt) below 1)^8^. If a large amount of transmissibility occurs in a small window of time, it is more difficult to identify the infected individuals before they transmit to more than one person^5^. We hypothesize that the interplay between an undetectable period during incubation and a non-uniform distribution of transmissivity leads to different outcomes for the efficacy of tests in combating disease spread compared to simple estimates of a uniform chance of transmission and a uniform false negative rate. To examine this, we developed a stochastic agent-based Susceptible-Exposed-Infectious-Recovered (SEIR) model of 10,000 students, roughly the size of the University of Maine. We find that the period of undetectable virus leads to a high basal apparent false negative rate, regardless of test sensitivity. When we consider the scenario where only testing is used to combat spread, we find that a simple model that assumes uniform viral spread and perfect tests predicts that testing everyone every 14 days may be sufficient to bring the Rt below 1. However, a model using the combination of disease spread based on the transmissivity data from He et al. and the dynamic false negative rates for tests from Kucirka et al. predict that as much as 100% of the population may need daily testing to bring the Rt below 1 and stop viral spread. While lower levels of testing can be effective in the presence of other interventions such as masking or social distancing, we conclude that the dynamics of an undetectable period, viral transmission that is biased early in the disease, and dynamic false negative rates significantly change the predictions of an SEIR model, and these factors should be considered when developing models to plan for public health interventions to combat COVID-19.

## Methods

### Model

**Figure 2:**
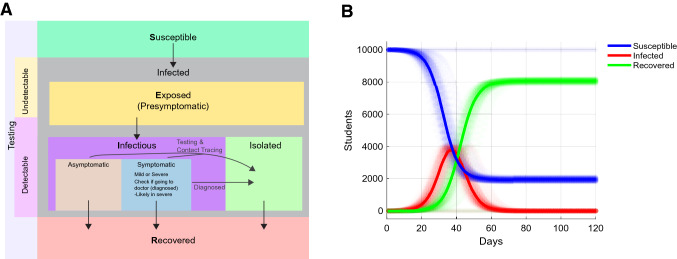
A stochastic agent-based model of COVID-19 transmission. This is a stochastic SEIR model implemented in MATLAB. Each transition in state is based on if–then statements with specific probabilities described in Fig. 3. (**A**) Individuals start as susceptible, and the initial population is seeded with 10 random infected individuals, each starting at a random point of progression through the disease, and with random symptoms. Upon being infected, an individual become exposed (presymptomatic), and is assigned a day for symptom onset. Detectability for testing and infectiousness both begin at 2 days prior to onset of symptoms. Infectious individuals can be either asymptomatic, or symptomatic with mild or severe symptoms. Those with severe symptoms will self-isolate and initiate contact tracing through seeking medical attention. Asymptomatic individuals and those with mild symptoms can be isolated through contact tracing or through detection by a test. Infectious individuals will recover randomly with a median time of 14 days. (**B**) An example of 100 independent simulations with the model. Shown are susceptible, infected (encompassing exposed, infectious, and isolated individuals), and recovered individuals in simulations where no interventions were implemented. Each individual simulation is represented as semi-transparent points, while the median value of all simulations is plotted as a line.

We chose to build a stochastic agent-based model for two reasons: (1) it would allow us to easily implement nonuniform probabilities over the course of infection and (2) a stochastic model would capture the inherent noise in a system that is presumed to start with a small number of infected cases. We implemented the model in MATLAB using the indicated probabilities and if–then statements. The test was performed with 10,000 individuals to represent the college student body. The model runs daily for 120 days, approximating a semester. The basic structure of the model is outlined in Fig. 2A. Because it is a stochastic model, we perform 100 independent runs (Fig. 2B), and report the median and 95th percentile results. The model can be found on GitHub at https://github.com/Kelley-Lab-Computational-Biology/coronamodel.

### Symptoms

**Figure 3:**
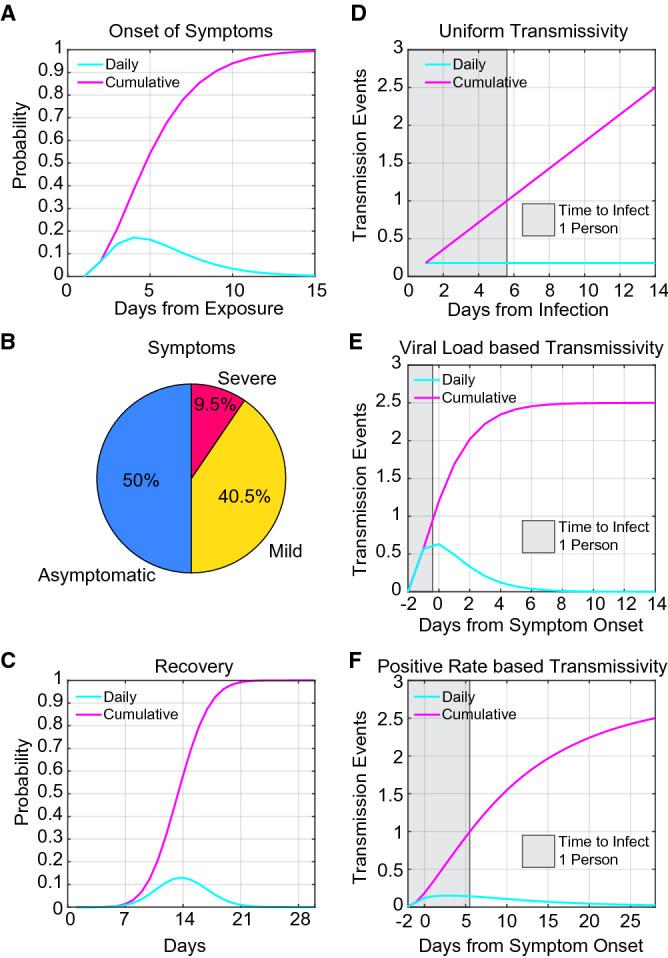
Model Parameters. (**A**) Probability distribution of onset of symptoms from He et al.^6^ (**B**) Breakdown of symptom groups in the model. (**C**) Probability distribution of recovery based on a median time to recovery of 14 days. (**D**) R0 of 2.5 scaled to a uniform transmission probability distribution. The gray box indicates where the cumulative probability reaches 1. Individuals must be detected prior to this, on average, in order to reduce the apparent R0 below 1. (**E**) The R0 of 2.5 scaled to the viral load based on He et al. The gray box is the same as above. (**D**) The R0 of 2.5 scaled to the positive test rate from Kucirka et al.^7^. This was done because the changes in positive test rate are likely related to viral load, and so may be an alternative representation of transmission likelihood. The gray box is the same as above.

For the timing of symptom onset, we used the symptom onset distribution calculated by He et al. This distribution has a median onset time of 4.2 days, and 99% of cases experience symptom onset by 14 days (Fig. 3A)^6^. An update to the He et al. paper considers an altered time to symptom onset (~ 6 days on average)^9^, but does not lead to significant differences in the model output (Supplemental Figure S1).

The CDC reports an overall asymptomatic rate of 40%^10^, but we are concerned about the likely asymptomatic rate among a young population. When the aircraft carrier Theodore Roosevelt had an outbreak of COVID-19, they reported that as many as 350 out of 600 sailors were asymptomatic for an asymptomatic rate of 58%^11^. As the population aboard a navy ship are likely to skew younger and healthier than the population as a whole, we felt they may be more representative of college age students. Thus, we assumed an asymptomatic rate of 50%. People who are symptomatic are then assigned either mild or severe symptoms, based on CDC data that 81% of people experience mild symptoms, 14% severe, and 5% critical^12^. We consider severe and critical together, as we expect both to seek medical assistance, and then be isolated from the general population. We also assumed that these numbers represent the percentages of symptomatic people, so ultimately the model assigns 50% asymptomatic, 40.5% mild, and 9.5% severe (Fig. 3B). We assume that those experiencing severe symptoms seek medical attention at the beginning of symptom onset and are isolated, and initiate contact tracing. Unless explicitly stated otherwise, we assume that mild cases do not self-isolate, as they may not realize that their symptoms are COVID-19 related^13^, or they may be reluctant to identify themselves as ill for fear of isolation and removal from their normal college activities^14^.

### Recovery

The CDC reports median recovery time as 14 days for mild illness^12^. We assume the same recovery period for asymptomatic people. Because severe illness results in medical attention and isolation, we did not consider the extended recovery period for severe illness as it would not change transmission in our model. The daily recovery probability is modeled as a binomial distribution with a mean of 14 days (Fig. 3C).

### Probability of viral spread

The model assumes an R0 of 2.5^10^. Each individual in the model receives an R0 normally distributed around 2.5 (with a standard deviation of 1) to allow for variability in transmissibility between people. We treat the population as well-mixed, and so when a transmission event occurs, it has an equal probability of infecting any individual in the population. If the randomly selected individual is isolated or recovered, then no infection takes place; only susceptible individuals are infected. For the normally distributed R0, we took three different approaches to viral transmission probability. (1) We assume a uniform daily transmission probability equal to 2.5/14 (R0 / median time of illness) (Fig. 3D). (2) We assume that daily transmission rate follows the transmission dynamics estimation from He et al.^6^, where transmission starts 2 days prior to symptom onset (Fig. 3E). An update to the transmission estimations from He et al. was published by the authors^9^ that changes the daily transmission probabilities but maintains the 44% presymptomatic transmission. We compared the effect of using the newer profiles in our model and found that they had a minimal impact on the predictions (Supplemental Figure S1) and so we continue to use the original profile published in^6^. (3) A daily transmission probability scaled to the false negative test rate reported by Kucirka et al.^7^, under the assumption that the dynamics of the false negative rate are related to the viral load (Fig. 3F). For each of these assumptions about viral spread, people must be detected on average before they spread virus to one other person (Rt below 1). We have indicated in Fig. 3 D,E,F with a shaded rectangle the time in which sick individual must be detected to keep the average number of new infections below 1.

We also explored the potential impact of super-spreader events on the outcome of the model, as superspreading is thought to be a strong driver of COVID-19 transmission^15,16^. In this case, we generated a distribution of R0s that leads to 20% of individuals causing 80% of transmission, following the 80/20 rule (Fig. 8)^17^ Each individual in the superspreader model has 35 interactions a day to allow for multiple transmission events, while using 1/35 of their daily transmission probability for each.

### Testing

Tests can be administered to the entire population, or to randomly selected subsets of the population either daily or at varied frequencies. Unless test delay is explicitly mentioned, we assumed tests are resolved on the day they are administered. We consider a few scenarios for false negative rates: (1) Perfect tests, where there is no chance of a false negative rate, and there is no period of undetectable infections. (2) Our “simple” scenario where the virus is undetectable until 2 days prior to symptom onset, after which tests have a uniform 5% false negative rate. (3) Dynamic false negative rates based on those measured by Kucirka et al.^7^. Like the simple scenario, there is no chance of detecting an infected individual prior to 2 days before symptom onset. We do not consider the ramifications of false positive rate. While the false positive rate is important due to the burden that incorrectly identified cases place on resources^2^, that consideration does not affect the Rt of the system. Tests work equally well on individuals who are symptomatic or asymptomatic in our model. The only difference in asymptomatic individuals is that they cannot self-isolate, they otherwise have a disease progression and viral load dynamics as if they were symptomatic. Testing of a fraction of the population was done by random sampling without replacement at the indicated frequency. Thus the identity of tested individuals is independent of previous tests, and no individual is tested more than once per day of testing.

### Contact tracing

For each individual in the model, we store the identity of the source of their infection, and the identities of people they transmit to. If someone is identified as sick by self-isolating and seeking medical attention, or if they are identified by a randomly administered test, contact tracing is initiated. We assume a 75% chance to identify each infected contact of the individual, as this is sufficient for contact tracing to work^18^, but not overly optimistic about the ability to find transient contacts in a University setting. Contacts that do not lead to infection are not explicitly modeled, thus the only contacts recorded in the model are transmission events. For each transmission event, the model determines if the individual is detected by the contact tracing success rate (75%) and then successfully identified contact will be moved to the isolated pool without the requirement for a positive test.

## Results

### Nonuniform false negative rates can delay detection of infected individuals

**Figure 4:**
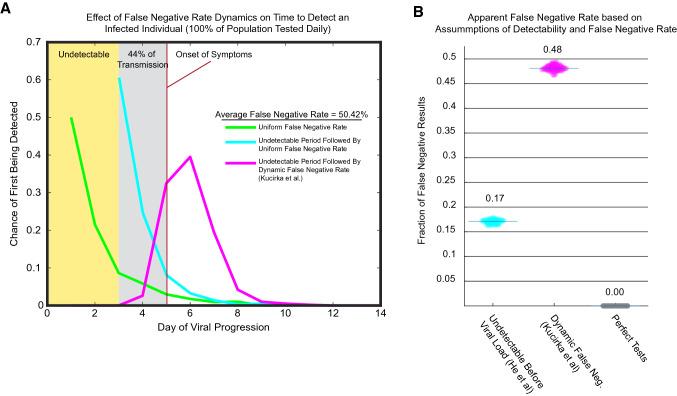
Effects of False Negative rates on detection. (**A**) Non-uniform false negative dynamics can delay detection of infected individuals. Shown is the chance of first being detected at each day of disease progression based on three scenarios with the same average false negative rate across the 14 days shown, but different temporal dynamics. For this graph, we assume that symptom onset begins at day 5. In yellow is the undetectable period prior to 2 days before symptom onset. The two days before symptom onset is shown in gray. Viral load data suggests that as much as 44% of transmissibility may occur in these two days. The line represents histograms of the first day that an individual would be detected by a daily test with the given false negative rate dynamics. (**B**) The undetectable period and temporal dynamics of the false negative rate lead to high apparent false negative rates. The first day of the model was run 100 times with 10,000 sick individuals. In cyan we show the model run with the simple assumption that infected individuals were undetectable before viral load begins, (2 days prior to symptom onset, based on the He et al. data^6^), and that after that point the tests will always detect infected individuals. In magenta, the model uses the dynamic false negative rates from Kucirka et al.^7^, in which both test error and inability to detect due to low viral load are mixed together. Also included as a comparison is the effect of perfect tests shown in gray.

Why are we concerned about uniform versus nonuniform false negative rate? To illustrate the issue, we can examine the first day of disease progression at which an infected individual is likely to be detected when tested daily. To examine the effect of detectability, we explore a hypothetical population where every person is sick and is tested daily (not the full SEIR model). We compare three different false negative rate dynamics over 14 days of disease progression assuming testing everyone, every day, and we assume symptom onset at day 5. The average false negative rate of each is the same (50.42%, chosen to match the overall average false negative rate of the Kucirka et. al. data^7^), but the way the rates change over time differs (Fig. 4). We have indicated with a gray rectangle the two days prior to symptom onset that may represent as much as 44% of viral transmission capability^6^. (1) A completely uniform false negative rate leads to most infected people being detected by day 3, prior to becoming infectious. (2) An undetectable period followed by a uniform rate of detection catches most individuals by day 5 (it is, after all, just a two day offset of (1), with the uniform rate rescaled to still average to the same overall false negative rate). These assumptions about the dynamics of viral spread allow more people to spend time in the infectious period prior to being detected than the completely uniform assumption. (3) The dynamic false negative rates of Kucirka et al. means that few individuals are likely to be caught prior to the potential for significant viral spread^7^.

### An undetectable period leads to high apparent false negative rates

The data from He et al. suggests that viral load reaches its maximal level prior to symptom onset and the transmission pair data suggest that virus first rises to a detectable level two days prior to symptom onset. Since viral RNA is the template for PCR based tests, the ability of the tests to detect the virus will be dependent upon the viral load, so we made the simple assumption that virus was undetectable prior to 2 days before symptom onset, and that it was uniformly detectable after this point. The measured false negative rates reported by Kucirka et al. validate this assumption, and provide daily false negative rates after viral load begins increasing. We made a separate model using the Kucirka et al. measured false negative rates.

We used the model to test the effect of these different assumptions on the overall false negative rate that would be encountered during testing for the virus on the first day of classes, where the people who are positive are randomly distributed (i.e. they have contracted the virus through independent events) through the progression of the disease. For example, while the median of symptom onset is between 4 and 5 days, 12% of cases would have a start of symptoms at 9 days or later. In this case, there would be at least 7 days during which there is insufficient virus present to detect an infection, regardless of the efficacy of the test. Simulations of the first day of the semester were run 100 times, and the median value of the false negative rate is reported (Fig. 4B). We found that the simple model, which assumes uniformly perfect tests after 2 days prior to symptom onset displays an apparent false negative rate of 17%. In the case of the Kucirka data^7^, which has both the undetectable time period before virus replication begins and the measured daily false negative rates afterward, which reach a minimum of ~ 25% two days after symptom onset, the overall false negative rate of the simulation was 48%. It is worth reiterating that this is the false negative rate one would experience testing a random group of people, not the false negative rate expected for directed testing, such as testing someone who is symptomatic. The Kucirka et al. false negative data is a compilation of both the false negative rate of the test, and the false negative rate due to the viral infection dynamics^7^. The simple model considers only the false negative rate from the viral dynamics and places the lower bound at 17% false negative, which is large but within the realm of consideration^2^.

### An undetectable period and high early transmission levels lead to a need for higher levels of testing

If the effective false negative rate ranges from 17% to as high as 48%, it is likely to affect the level of testing required to combat the spread of COVID-19. We set out to examine the effect of testing on the spread of disease by calculating the effective R0 of the virus when different testing regimens are used, while varying the dynamics of detectability and test false negative rate. We varied the fraction of the population being tested and the frequency of the test for four scenarios. For random testing of a fraction of the population, each person can only be tested once per testing and the sampling for testing on different days is independent of previous tests, such that a given individual may be tested multiple times in a row or may be skipped. The four different viral dynamics scenarios are:

*Scenario 1: “Perfect tests, Uniform Spread”* where we assume no period of undetectability, no false negative rate, and a uniform chance of transmission equal to 2.5 / 14.

*Scenario 2: “Simple Undetectability, Fast Spread”* where we assume that the virus is not detectable until 2 days prior to symptom onset, and then has a 5% false negative rate after that point (this 5% false negative rate is a change from the simple assumption above (Figure 6), which assumed perfect tests). This condition uses the He et al. viral load data to scale the R0 (Figures 1 and 4E), which results in ~45% of transmissivity prior to symptom onset.

*Scenario 3: “Dynamic False negative, Slow Spread.”* This uses the day-by-day false negative rates reported by Kucirka et al. for testing (Figure 1). For transmissivity, we use the day-by-day positive rates from the Kucirka et al. data as a stand-in for viral load (Figure 4F). The shape of this profile still biases spread early in the disease, but not as early as the He et al. viral load data.

*Scenario 4: “Dynamic False negative, Fast Spread.”* This scenario uses the day-by-day false negative rates from Kucirka et al. for testing, and the He et al. transmissibility estimates.

**Figure 5:**
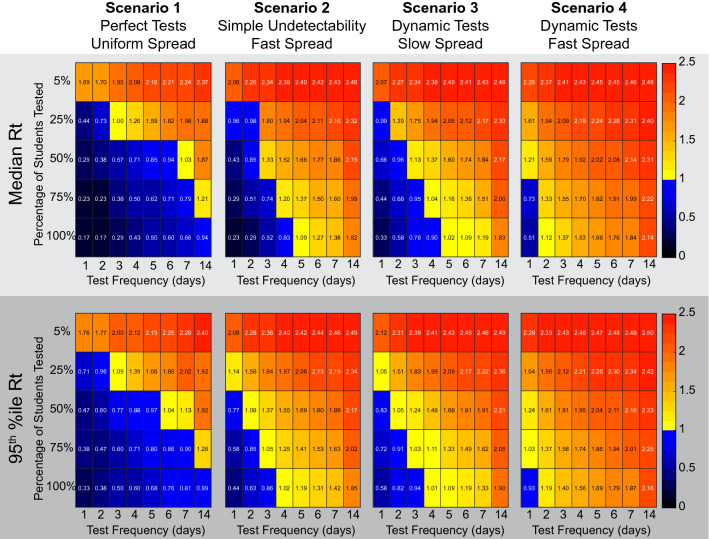
High asymptomatic transmission and dynamic false negative rate lead to a requirement for more testing to bring the viral spread under control. Heatmaps show the effective Reproduction number (Rt) from 100 simulations run with the given proportion of the population tested at the indicated frequency. The top row of matrices shows the median Rt, while the bottom row of matrices shows the value of the upper 95th percentile (i.e. conditions that will work in 19 out of 20 situations). While the scenario 1 perfect tests suggest testing the entire population every two weeks may work to stop spread of the virus, using scenario 4 parameters predicts that testing the entire population daily was necessary.

These simulations are run with testing and subsequent isolation of positives being the only intervention used to combat viral spread. We report the median effective Rt as well as the 95th percentile Rt for each condition because testing regimens that work only half the time may not be useful when considering public health. We see that perfect tests can be effective while testing as little as 25% of the populace every other day (Fig. 5). All simulations that do not assume perfect tests require a larger proportion of the population to be sampled under these conditions. Scenario 2 and Scenario 3 result in remarkably similar results for which testing regimens are required for suppression of viral spread. The fast viral-spread and sensitive tests of Scenario 2 are therefore compensated for by the slower viral spread and insensitive testing of Scenario 3. With scenario 4, where the transmission occurs early in the disease and false negative rates are high, only testing of every individual every day was able to bring the Rt below 1. Thus, viral transmission that is biased early in the progression of the disease and higher false negative rates require a more aggressive testing regimen than would be suggested by uniform assumptions.

**Figure 6:**
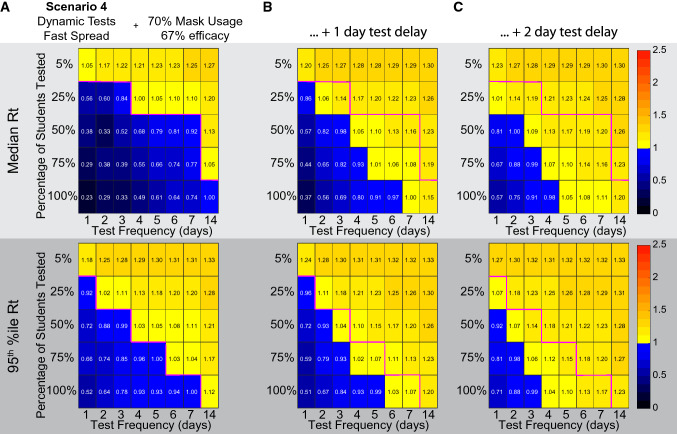
In the presence of masking, fewer tests and lower frequencies of testing can be successful in driving Rt below 1. (**A**) Here we implemented 70% of the population using a mask that is 67% effective with the parameters of Scenario 4, early transmission of virus based on the He et al. viral load data, and dynamic false negative rates for tests based on Kucirka et al. The top row of matrices shows the median effective Reproduction number (Rt), while the bottom row of matrices shows the value of the upper 95th percentile. Masking drove the median Rt from 2.5 to ~ 1.3. Tests were then able to drive the 95th percentile Rt below 1 with less aggressive testing schemes than in Fig. 5. (**B**) The same conditions as (**A**), with an included 1 day turn around delay in testing results. The magenta line shows the border between an Rt above 1 and an Rt below 1 without a delay. (**C**) As in (**B**) with a 2 day turn around delay in testing results.

While these simulations suggest that testing would have to be very aggressive to bring viral spread under control, they are not assuming any interventions beyond testing followed by contact tracing and isolation. In reality, testing is likely to be a component of a multi-pronged approach to combating viral spread that would include social distancing and masking. We decided to examine the efficacy of testing under a situation where other interventions had brought the viral spread down, but not below an Rt of 1. A recent study of mask efficacy suggests that surgical or cloth mask wearing can reduce the risk of contracting COVID-19 to 33% the risk of those not wearing masks^19^. Interestingly, this is similar to the percent decrease in particulates that has been described for a cloth mask^20^ (average reduction in particulates to ~ 31% of control over a 3 h experiment). We implemented a model where 70% of the population uses masks that reduce transmission rate by 67%. This results in a median apparent Rt of 1.3, and a 95th percentile value of 1.44. We then performed the simulations using the array of testing regimens as above. For this analysis, we used the Scenario 4 conditions of dynamic false negative rate^7^ and high early viral transmission dynamics^6^, as these conditions are the hardest to reduce and will give the most conservative results for frequency and amount of testing. We find that under these conditions it would now be possible to bring the Rt below 1 in 95% of cases by testing 25% of the population every day (Fig. 6A). Testing every person would now be effective when done once a week.

These previous simulations assume instantaneous turnaround time for the test results. Unfortunately, test results may take a day to several days for results to be available. The delay is typically due to the backlog of samples needed to be tested, lack of testing equipment, and the relatively small number of labs and technicians with proper certification^21^. To analyze the effect on delay in receiving the results, we used Scenario 4 conditions with 70% of the population using masks then performed the simulations using the array of testing regimens as above. In the model, students who are tested and found positive start their isolation after they find out their results along with people isolated due to contact tracing. Figure 6B shows how implementing a one-day delay has a detrimental effect on the testing requirement in order to prevent an outbreak. A one-day delay in receiving test results leads to a requirement for a two-day increase in frequency, as testing the whole population every 5 days would prevent an outbreak compared to testing every 7 days with no delay. Similarly, a two-day delay in receiving test results leads to a four-day increase in testing frequency necessary to prevent an outbreak (Fig. 6C). The delay in test results significantly changes the testing frequency requirements in order to prevent an outbreak.

**Figure 7:**
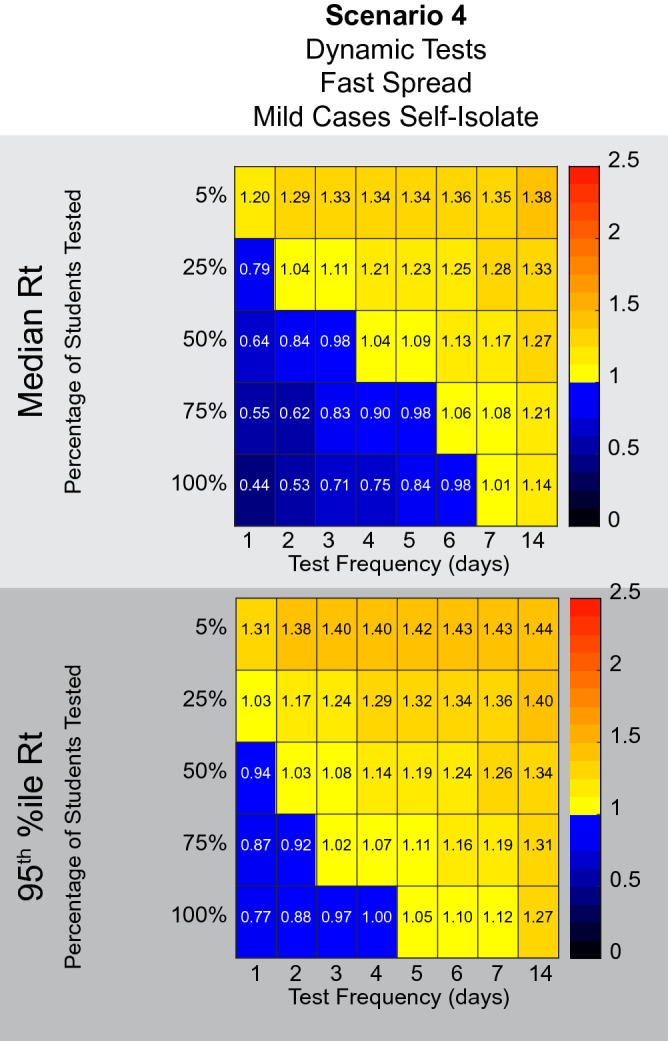
Assumptions about the behavior of people with mild symptoms influence the amount of testing required. Scenario 4 from Fig. 6 was repeated with the addition that people experiencing mild symptoms self-isolate on the day of symptom onset. Under these conditions, testing everyone every 4 days is sufficient to bring 95% of situations to an Rt of 1.00 or below.

The degree to which people with mild symptoms will decide that they have COVID-19 instead of dismissing their symptoms as insignificant is unclear. For example, a traveler was originally identified as an asymptomatic spreader early in 2020, when in fact she had symptoms at the time but did not identify them as severe enough to consider herself sick^13^. Ultimately, she had severe COVID-19 and was even hospitalized. Presumably, those with moderate symptoms may also have difficulty differentiating between symptoms indicating COVID-19, or something innocuous, such as seasonal allergies. Additionally, a survey of students has suggested that they may be reluctant to remove themselves from school for mild symptoms^14^. For these reasons we assumed that people with mild symptoms did not self-isolate in the above simulations. To examine this issue, we tested how self-isolation by people with mild symptoms would affect the necessary testing regimen to contain spread using Scenario 4 conditions as above. Under circumstances where people are very responsible and self-isolate under any mild symptoms of illness, we see that testing 100% of the population every 4 days becomes sufficient to successfully bring the Rt to 1.00 or below in 95% of simulations (Fig. 7). Thus, the degree to which the population of interest takes seriously the instructions to self-isolate if they are feeling ill could influence the efficacy of any given testing strategy and could allow less stringent testing plans to succeed. The frequency of self-isolation due to perceived symptoms is a parameter that this model is very sensitive to (Supplemental Figure S2), and a parameter that may vary significantly from one population to another.

### Superspreader events increase the variability in the spread of COVID-19

**Figure 8:**
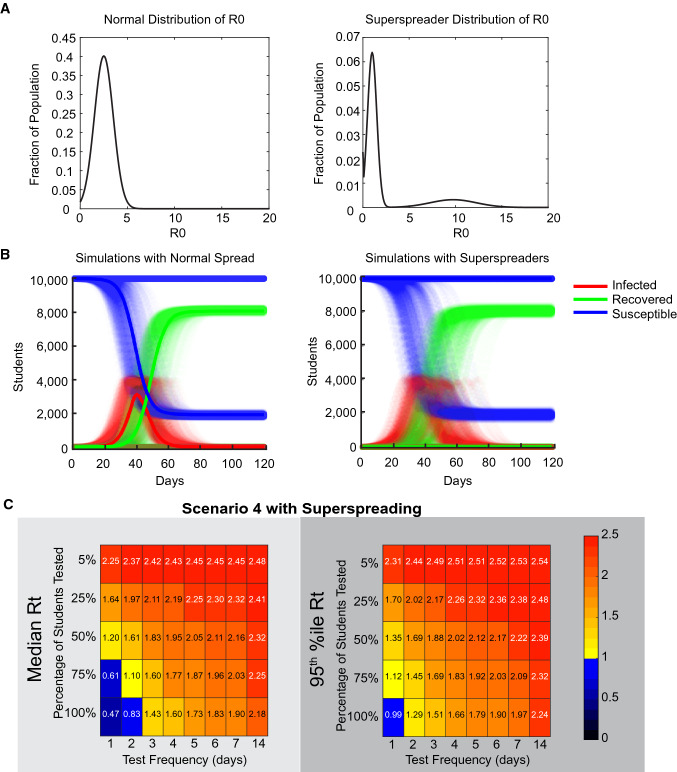
Superspreading leads to more variability between discrete simulations, but requires the same amount of testing as normally distributed transmission probabilities. (**A**) On the left is the normal distribution of R0s assigned to individuals, while the right distribution is a superspreader distribution where 20% of the population is responsible for 80% of the infections. (**B**) 500 runs of the model with either the normal distribution or the superspreader distrubtion of R0s and the Scenario 4 transmission and false negative rate dynamics. Medians of each population are shown as solid lines. Note that the medians of infected and recovered in the superspreader conditions fall on the x-axis line. (**C**) Effective reproduction number (Rt) for the indicated testing frequency and proportion of population for the superspreader R0 distribution. As in Fig. 5, only daily testing of 100% of individuals is sufficient to drive Rt below 1 under these assumptions.

Superspreading events, where one individual transmits virus to many more individuals than one would expect on average, seem to play a large role in the spread of COVID-19^15^ and it has been estimated that 5% of people may be responsible for as much as 80% of cases^16^. In our model, we approach variability in transmission between individuals by assigning each individual their own R0, a constant for that individual’s chance to transmit, rather than giving each person the same R0 and differing numbers of opportunities to transmit. To examine the effect of this phenomenon on the requirement for testing, we generated a superspreading probability distribution where 20% of sick individuals were responsible for 80% of cases, following the general 80–20 rule^17^ (Fig. 8A), which leads to most people having little to no transmission, and a small number of people being highly transmissible. 500 simulations were run with a first day test of all students and the Scenario 4 transmission and false negative rates for both R0 distributions (Fig. 8B). Incorporating superspreaders into the model leads to more variability in the timing of outbreaks and an increase in the number of situations where no disease occurs (lucky testing on day 1, or contagious individuals who do not end up transmitting). In those cases where outbreaks occur, they lead to the same level of illness, as one would expect for models with the same R0. When we examine testing regimen, we find that median Rt values do change a bit, (due to the increased frequency of no transmission) but that the testing required to stop 95% of outbreaks remains the same as that found for Scenario 4 in Fig. 5. Thus, superspreading increases the variability in outcomes for situations. We may have seen superspreading driven variability between discrete situations borne out in the real-world differences that were seen between COVID-19 occurrence on different college campuses in the fall of 2020^22^.

## Discussion

Available data on SARS-COV2 viral load over time and on false negative rate of tests over time both suggest that virus may not be detectable prior to ~ 2 days before symptom onset, and transmissibility of the virus is biased towards the beginning of disease progression. Here we have examined the effect of nonuniform viral transmission and nonuniform detectability of disease on the efficacy of testing as a means to stop viral spread. We find that the combination of the non-uniform transmission dynamics and false negative rate predict that tests must cover more of the population and be given more frequently than predicted by a model that assumes uniform distributions. Thus, models that make simple assumptions about viral spread, and false negative rate or underestimate the effect of the undetectable period on the apparent false negative rate may recommend less testing than is necessary to stop viral spread.

The parameters used for these simulations (transmissibility dynamics, false negative rate, voluntary self-isolation, efficacy of masks and level of compliance with masking) are not concrete, and are likely to vary between institutions, populations, or areas. As the model parameters approach containment of viral spread, the prevalence of virus in the surrounding community, or other sources of introduction into the system will be more important to the considerations for testing amount and frequency as well as quick turnaround time of results. As such, these results should not be seen as concrete recommendations on specific testing strategies, although the results for Scenario 4 are clearly conservative, and others have come to similar conclusions about the need for frequent testing^23,24^. Similarly, these studies should not be construed as saying that tests do not work or that tests should not be a part of the public health strategy for combating viral spread. Instead, the takeaway message is that modeling of tests should be done with consideration of the potential for an undetectable period, nonuniform transmission dynamics, and the potential for viral load to influence false negative rate. Each of these considerations alters the conclusions that a model will come to about the number and frequency of tests required to combat viral spread.

There are many reports in the news media of organizations using a negative test result as a prerequisite for engagement in some activity, such as returning to college or attending a summer camp. The Kucirka et al. data on dynamic false negative rate should already give pause to these types of plans, but we show here that testing a population of people who may have a random distribution of progression through disease may have a false negative rate as high as 48%. The possibility of missing ~ 1/2 of positive individuals by performing a complete testing of the population of interest should be considered when making these plans. This high false negative rate is specific to tests that are performed on a population likely to have a random distribution of viral progression. In situations where the tests are being given because of symptoms, or because of contact tracing, the population being tested would be biased towards later days in the progression of the disease, and the overall false positive rate would be lower than the 48% value. However, even if one were using a test that was 100% sensitive and specific given a sample that contains template, it is likely that they would still experience the ~ 17% false negative rate due to the latent period of the virus before it reaches sufficient levels to be detectable. Thus, plans to allow people to participate in activities dependent upon a negative test should be aware of the greater than 1 in 6 likelihood of missing an infected person in their testing.

In conclusion, many people are resorting to modeling of disease transmission to assist in the formulation of public health plans for the return to schools and economic activities. When designing these models, simple assumptions of uniformity of transmission and uniformity of false negative rate can give overly optimistic views of the efficacy of testing. These nonuniform dynamics are complicated to implement in a deterministic ODE model, but easier to implement in a stochastic agent-based model. The stochastic model, however, is slow compared to an ODE model. Answering questions about tests does not, however, require a population to be so large as to be unmanageable with a stochastic model, as the trends in testing efficacy should remain the same. Thus, we recommend that stochastic models be used to model efficacy of tests so that complex dynamics can be readily accounted for. The results of stochastic models could then be used to parameterize deterministic models for other uses.

## Supplementary Information

Supplementary Information
