# Supplementary material for: Temporal dynamics of viral load and false negative rate influence the levels of testing necessary to combat COVID-19 spread
Source: Sci Rep. 2021 Apr 28;11:9221. doi: 10.1038/s41598-021-88498-9 (PMC8080800; doi:10.1038/s41598-021-88498-9)
Supplement: Supplementary file 1 — Supplementary Information [file 41598_2021_88498_MOESM1_ESM.pdf]

Supplemental Figure S1

A)

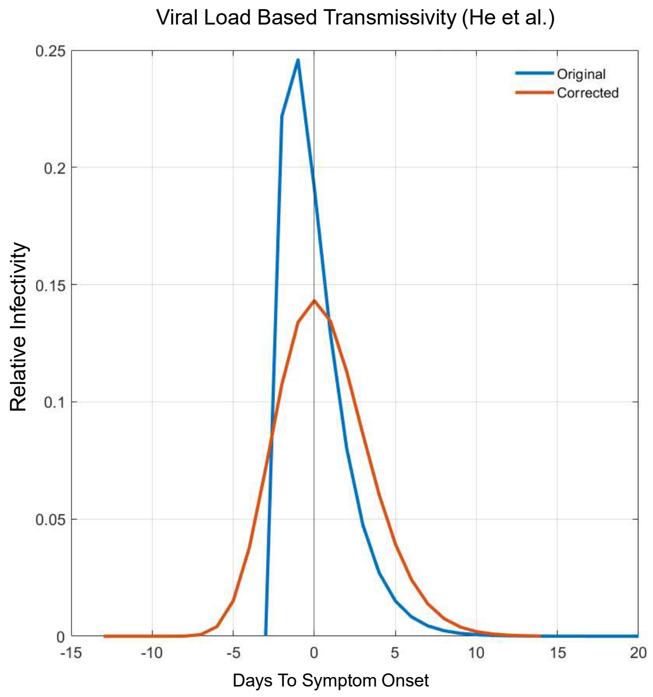

B)

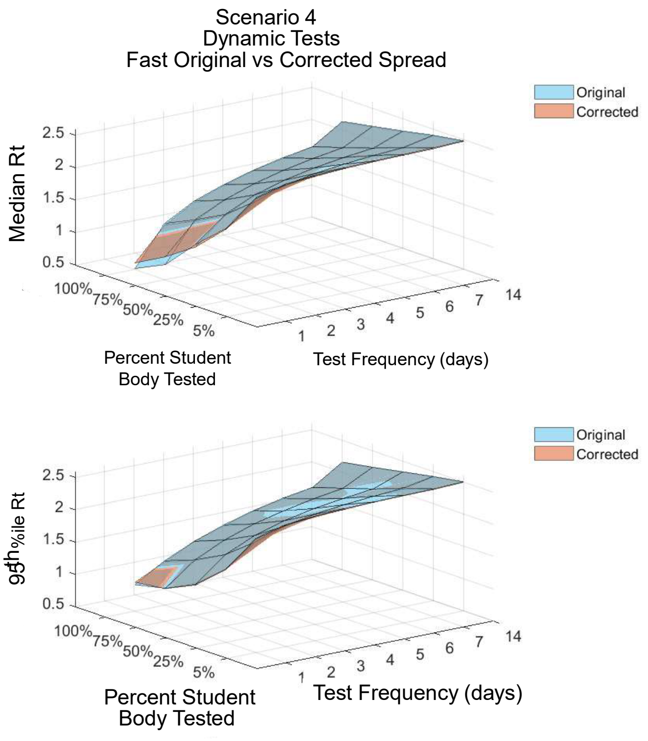

Supplemental Figure 1: The Effect of He et al. Updated Transmissivity on Rt
A) The original He et al. transmission probability [6] (blue) compared to the corrected values [13] (red) in respect to the day of symptom onset. B) The new viral load distribution was incorporated into the Scenario 4 model and the effective Reproduction number (Rt) was calculated from 100 simulations run with the given proportion of the population tested at the indicated frequency. The results from the Corrected Scenario 4 were compared to the Original Scenario 4. The top plot compares the median Rt, while the bottom plot compares the bootstrapped value of the upper 95th percentile Rt.

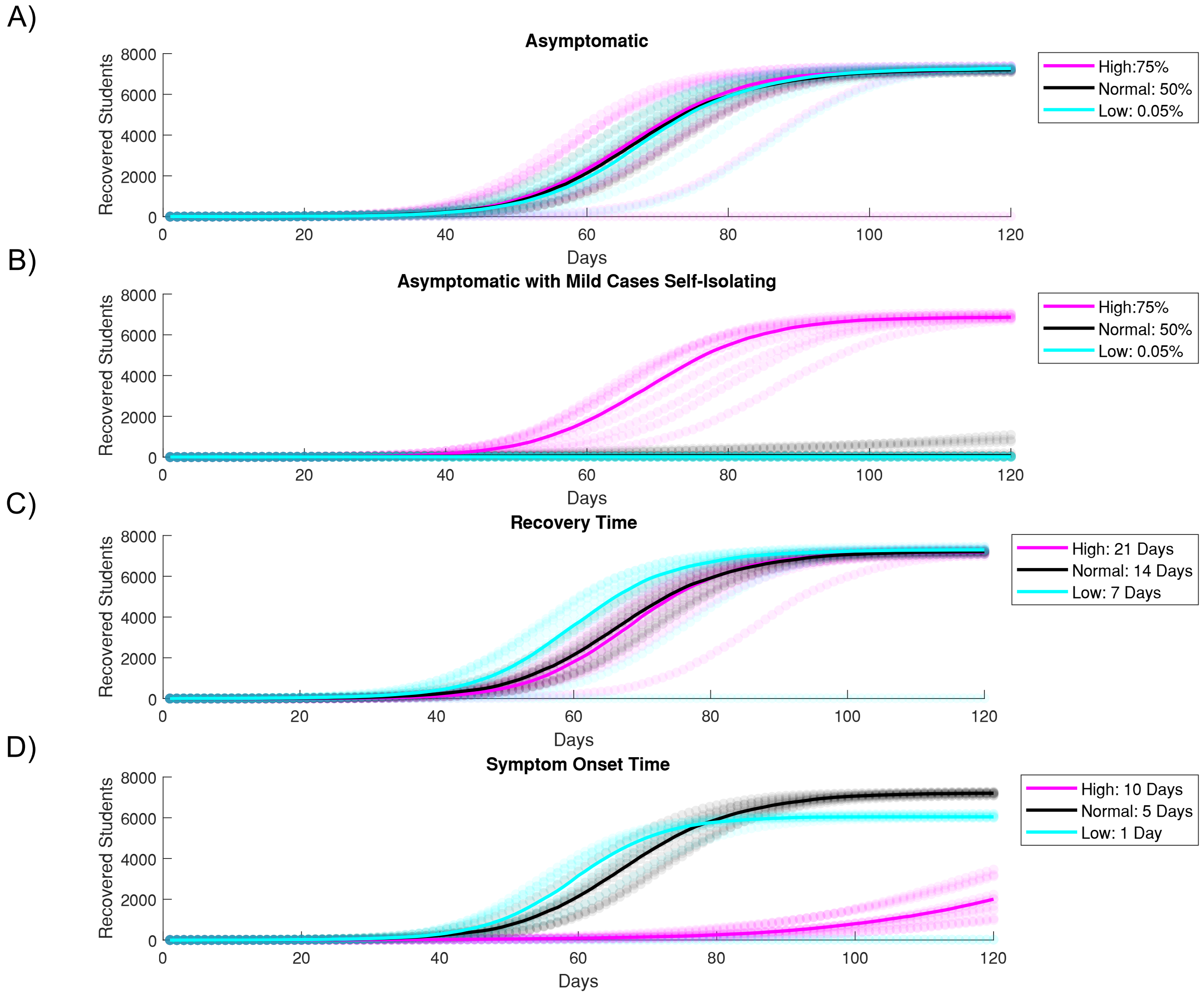

Supplemental Figure S2. Sensitivity Analysis of Parameters. A sensitivity analysis was performed by altering parameters to be less than (cyan), equal to (black) and greater than (magenta) the parameter used in the model. The altered parameters were tested by running Scenario 4 with 10 replicates for each altered parameter. The results were compared by looking at the number of recovered students in each iteration (dotted line) as well as the median recovered students (solid line). A) The parameter of asymptomatic cases was tested at 5% and 75% to compare to the normal 50% of cases. B) The parameter of asymptomatic cases with mild cases self-isolating was tested at 5% and 75% to compare to the normal 50% of cases. C) The parameter of recovery time was tested at 7 days and 21 days to compare to the normal 14 days recovery time. D) The parameter of symptom onset time was tested at 1 days and 10 days to compare to the normal 5 days symptom onset time.
